# Sequential Bilateral Central Retinal Vein Occlusion With Differential Long-Term Outcomes Following Cardiac Surgery

**DOI:** 10.7759/cureus.100045

**Published:** 2025-12-25

**Authors:** Toshihiko Matsuo, Zenichi Masuda, Hiroki Sugiyama, Kazufumi Nakamura

**Affiliations:** 1 Ophthalmology, Graduate School of Interdisciplinary Science and Engineering in Health Systems, Okayama University, Okayama University Hospital, Okayama, JPN; 2 Cardiovascular Surgery, Okayama University Hospital, Okayama, JPN; 3 Cardiovascular Medicine, Okayama University Hospital, Okayama, JPN; 4 Cardiovascular Medicine and Center for Advanced Heart Failure, Okayama University Hospital, Okayama, JPN

**Keywords:** aortic valve regurgitation, aortic valve replacement, bevacizumab, bilateral central retinal vein occlusion, intravitreal injection, mitral valve annuloplasty, mitral valve regurgitation, panretinal laser photocoagulation, tricuspid valve annuloplasty, tricuspid valve regurgitation

## Abstract

Bilateral central retinal vein occlusion (CRVO) is rare and is associated with systemic diseases such as hypertension, diabetes, dyslipidemia, and coagulopathy. In this study, we showed that the sequential development of bilateral CRVO in an elderly patient was related to increased venous pressure in the right heart system. A 71-year-old man developed CRVO in the right eye, and one year later, he developed CRVO in the left eye. He had undergone pacemaker implantation for sick sinus syndrome 10 years earlier and had started hemodialysis three months prior for chronic renal failure, probably caused by hypertensive nephrosclerosis. The right CRVO resulted in neovascular glaucoma and loss of light perception despite intensive treatment with panretinal laser photocoagulation, intravitreal bevacizumab injection, and additional laser therapy. In contrast, the left CRVO remained at an impending stage, was treated only with panretinal laser photocoagulation, and had a favorable outcome for 11 years until his death. In retrospect, half a year after the onset of left CRVO, the patient underwent open-heart surgery to repair aortic, mitral, and tricuspid valve regurgitation through aortic valve replacement, mitral valve annuloplasty, and tricuspid valve annuloplasty, respectively. Based on the temporal sequence of events, elevated venous pressure due to right heart dysfunction may have contributed to the poor outcome of the right CRVO, whereas improvement of venous stasis after cardiac surgery may have led to the better long-term outcome of the left CRVO. Venous stasis in the right heart system should therefore be considered an underlying factor in the development of bilateral CRVO.

## Introduction

Retinal vein occlusion is a common ocular disease, particularly in elderly individuals [1,2]. In the ocular circulatory system, the ophthalmic artery, as the first branch of the internal carotid artery, gives rise to the central retinal artery, which penetrates the optic nerve and divides into four branches at the optic nerve head to supply the retinal tissues [3]. The retinal capillaries converge into four branches of retinal veins, which further converge to form the central retinal vein at the optic nerve head. The central retinal vein then exits the optic nerve, becomes the ophthalmic vein, drains into the cavernous sinus, and ultimately empties into the internal jugular vein and the superior vena cava. As a collateral route within the orbit, the ophthalmic vein also anastomoses with the angular vein, draining into the facial vein and subsequently into the internal jugular vein and the superior vena cava [3].

Retinal vein occlusion is classified into branch retinal vein occlusion and central retinal vein occlusion (CRVO). Retinal vein occlusion often occurs at arteriovenous crossing points, where a sclerotic arteriole with a hardened vascular wall compresses the adjacent retinal venule through mechanical pressure. Common risk factors for both branch and CRVO include aging, hypertension, diabetes, and dyslipidemia, all of which affect the vascular walls [4,5]. In addition, vein occlusions are generally attributed to alterations in blood flow induced by hypercoagulable states, hyperviscosity syndromes, venous stasis, and obstructive sleep apnea [6].

CRVO is less common than branch retinal vein occlusion and may be associated with systemic risk factors at a higher frequency, particularly in cases of bilateral involvement [4,5]. In this report, we describe an elderly patient who developed bilateral CRVO sequentially, first in the right eye and then in the left eye one year later. The CRVO in the right eye, which occurred first, was refractory to intensive treatment with panretinal laser photocoagulation and intravitreal bevacizumab injection, the current standard of care [7-10], and resulted in loss of light perception. In contrast, the CRVO in the left eye, which developed one year later, remained impending and had a favorable outcome following panretinal laser photocoagulation after the patient underwent open-heart surgery for tricuspid, mitral, and aortic valve regurgitation. Elevated venous pressure associated with right heart dysfunction may have contributed to the poor outcome in the right eye, whereas surgical repair leading to reduced venous pressure may have resulted in the favorable outcome in the left eye.

## Case presentation

**Figure 1:**
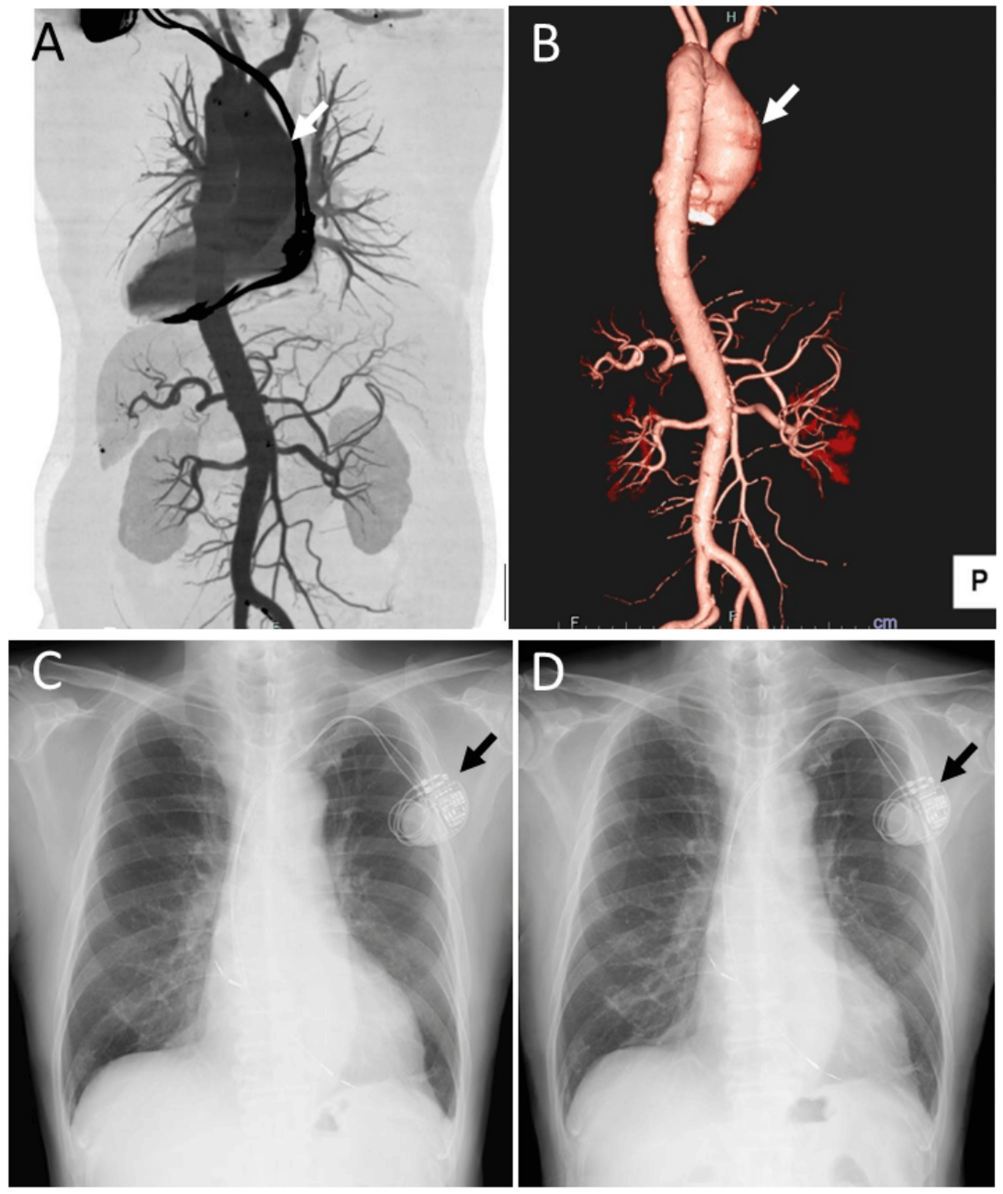
CT angiography at age 65 years and plain chest radiographs at age 71 years CT angiography (posterior views) at age 65 years (A, B) showing a fusiform ascending aortic aneurysm with a diameter of 51 mm (arrows). Plain chest radiographs at age 71 years, obtained one month before presentation (C) and at the onset (D) of right CRVO, showing no change in cardiomegaly on the two occasions. Arrows indicate the cardiac pacemaker.

A 71-year-old man noticed chromatopsia in the right eye for two weeks and was diagnosed with CRVO in the right eye by a local physician. His past medical history included a diagnosis of hypertension in his 30s and surgical resection of tongue cancer at 60 years of age, with no recurrence. A cardiac pacemaker had been implanted at 61 years of age for sick sinus syndrome, and he was diagnosed with a fusiform ascending aortic aneurysm measuring 51 mm in diameter. At 65 years of age, he experienced an episode of back pain, and CT angiography revealed no dissection or rupture of the aortic aneurysm (Figure 1A, 1B).

He was also noted to have chronic renal failure, likely due to hypertensive nephrosclerosis, although this was not confirmed by renal biopsy at the patient’s request (Table 1). He had smoked 20 cigarettes (one pack) daily for 45 years and subsequently reduced his smoking to five to six cigarettes daily until the present. He was an occasional alcohol drinker. His medications included oral furosemide 40 mg, telmisartan 40 mg, carvedilol 10 mg, and amlodipine 5 mg daily for hypertension, as well as warfarin 2 mg daily for atrial fibrillation. His blood pressure was 146 mmHg systolic and 78 mmHg diastolic. Physical examination revealed systolic and diastolic murmurs, but no jugular venous distention or lower limb edema. Plain chest radiography showed cardiomegaly of similar extent compared with a chest radiograph obtained one month earlier (Figure 1C, 1D). Three months prior to presentation, he had begun hemodialysis three times weekly.

**Table 1:** Blood examinations during the clinical course of the patient ^*^ High values due to hemolysis of the blood sample ALT, alanine aminotransferase; AST, aspartate aminotransferase; BNP, brain natriuretic peptide; CRVO, central retinal vein occlusion; eGFR, estimated glomerular filtration rate; LD, lactate dehydrogenase; n.d., not determined; NT-proBNP, N-terminal pro-B-type natriuretic peptide

Parameter/event	Normal range	61 years (just after pacemaker implantation)	65 years (none)	71 years (just before hemodialysis initiation)	71 years (at onset of right-eye CRVO)	72 years (at onset of left-eye CRVO)	73 years (just before heart surgery)	76 years (none)	80 years (none)	83 years (one week before death)
Red blood cells (×10⁶/µL)	4.35-5.55	4.57	4.57	3.08	3.92	3.6	3.6	3.03	3.47	2.4
Platelets (×10³/µL)	158-348	137	165	102	55	51	69	67	79	69
White blood cells (×10³/µL)	3.30-8.60	7.8	6.27	4.28	6.19	3.99	3.19	5.11	3.11	4.64
Hemoglobin (g/dL)	13.7-16.8	14.5	14.6	9.6	12.6	11.8	11.5	9.7	11.2	7.9
Hematocrit (%)	40.7-50.1	42.2	43	30.8	38.7	36.1	35.6	30.6	37.1	26.7
Total protein (g/dL)	6.6-8.1	5.77	6.4	6.2	6.9	6	5.9	5.8	5.6	4.2
Albumin (g/dL)	4.1-5.1	3.08	3.7	3.7	4.1	3.6	3.5	3.2	3	2.3
LD (U/L)	124-222	218	168	197	212	228	202	293	802^*^	236
AST (U/L)	13-30	18	19	11	15	22	25	18	54^*^	30
ALT (U/L)	10-42	14	23	11	14	25	17	8	10	10
Total bilirubin (mg/dL)	0.40-1.50	1.7	1.05	0.72	1.69	1.03	0.86	1.55	0.74	0.38
Urea nitrogen (mg/dL)	8.0-20.0	19.1	23.5	91.4	25.9	43.5	21.4	49.2	29.1	59.2
Creatinine (mg/dL)	0.65-1.07	0.98	1.47	8.39	4.52	6.42	3.99	7.81	3.64	7.24
eGFR (mL/min/1.73 m²)	≥60	n.d.	n.d.	5.6	11	7.4	12.5	5.9	13.4	6.3
Postprandial glucose (mg/dL)	<140	n.d.	173	126	95	119	n.d.	n.d.	180	n.d.
BNP (pg/mL)	0-18.4	n.d.	89.4	n.d.	709.2	1177	1136	1114.7	n.d.	735
NT-proBNP (pg/mL)	<125	n.d.	n.d.	n.d.	n.d.	n.d.	n.d.	n.d.	32,295	n.d.

**Figure 2:**
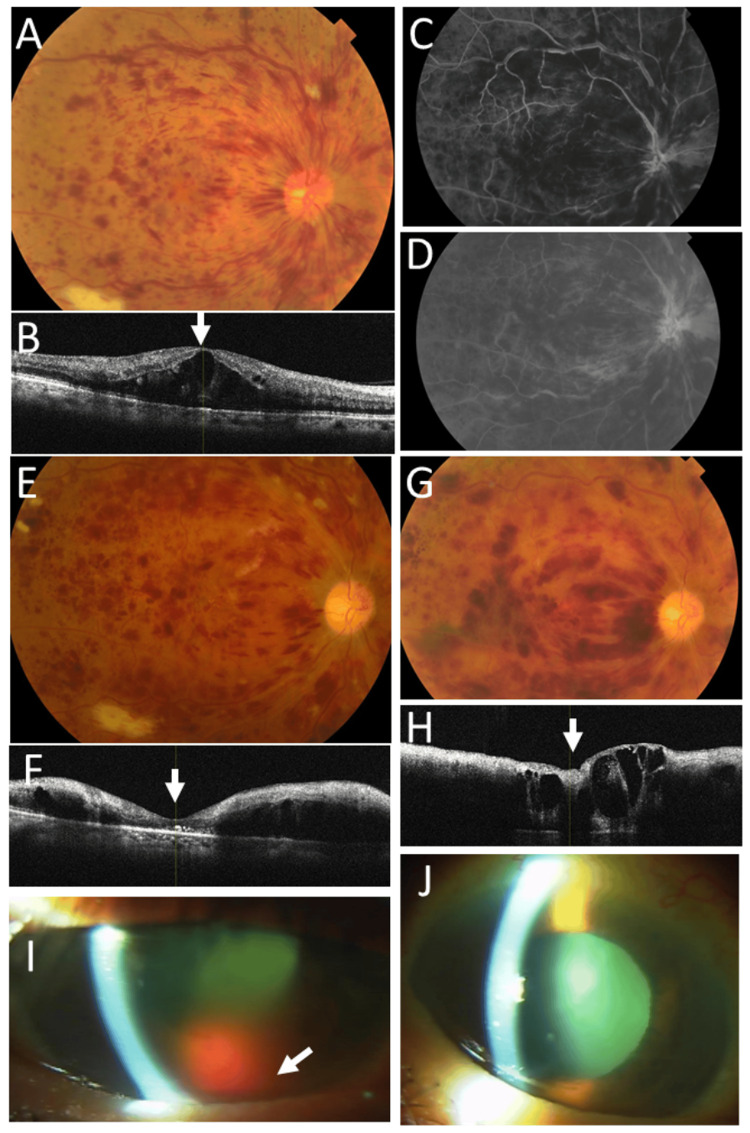
Ophthalmic images at age 71 years Fundus photograph (A), horizontal OCT section (B), and early-phase (one minute and 11 seconds; C) and late-phase (13 minutes and 42 seconds; D) fluorescein angiography images obtained at presentation of CRVO in the right eye at age 71 years. Note marked flame-shaped and blot retinal hemorrhages with retinal venous dilatation (A), macular edema (B), and delayed venous filling (C) and drainage (D). Fundus photographs and horizontal OCT images obtained one month (E, F) and four months (G, H) after the onset of CRVO in the right eye. Note resolution of macular edema one month after intravitreal bevacizumab injection and panretinal laser photocoagulation (arrow, F) and recurrence at four months (arrow, H). Slit-lamp photographs of the right eye (I) and left eye (J) obtained 10 months later show hemorrhage (hyphema) in the anterior chamber of the right eye (arrow, I). CRVO, central retinal vein occlusion; OCT, optical coherence tomography

At referral, best-corrected visual acuity (decimal notation) was 0.5 in the right eye and 1.0 in the left eye. Intraocular pressure was 10 mmHg in the right eye and 12 mmHg in the left eye. The right eye showed marked dilatation of retinal veins with numerous flame-shaped and blot retinal hemorrhages and macular edema (Figure 2A-2D), whereas the left eye was normal. Within a few days, visual acuity in the right eye decreased to 0.02, and the patient underwent an intravitreal injection of bevacizumab and panretinal laser photocoagulation in three weekly sessions in the right eye, followed by a second intravitreal injection of bevacizumab. This resulted in the resolution of macular edema in the right eye (Figure 2E, 2F).

Four months after the initial visit, macular edema relapsed with persistent retinal hemorrhages in the right eye (Figure 2G, 2H), and intraocular pressure increased markedly to 35 mmHg. Visual acuity in the right eye was 0.04. Gonioscopic examination revealed injection of Schlemm’s canal together with mild neovascularization of the iris at the iridocorneal angle. The patient underwent three additional sessions of retinal laser photocoagulation in the right eye. However, he developed hyphema in the anterior chamber of the right eye, resulting in visual acuity limited to hand motion. Intraocular pressure in the right eye was 16 mmHg with topical treatment using 0.005% latanoprost and a combination solution of 1% dorzolamide and 0.5% timolol.

Ten months after the initial visit, hemorrhage (hyphema) in the anterior chamber of the right eye persisted (Figure 2I). In contrast, the left eye remained normal (Figure 2J). Visual acuity was hand motion in the right eye and 0.9 in the left eye.

**Figure 3:**
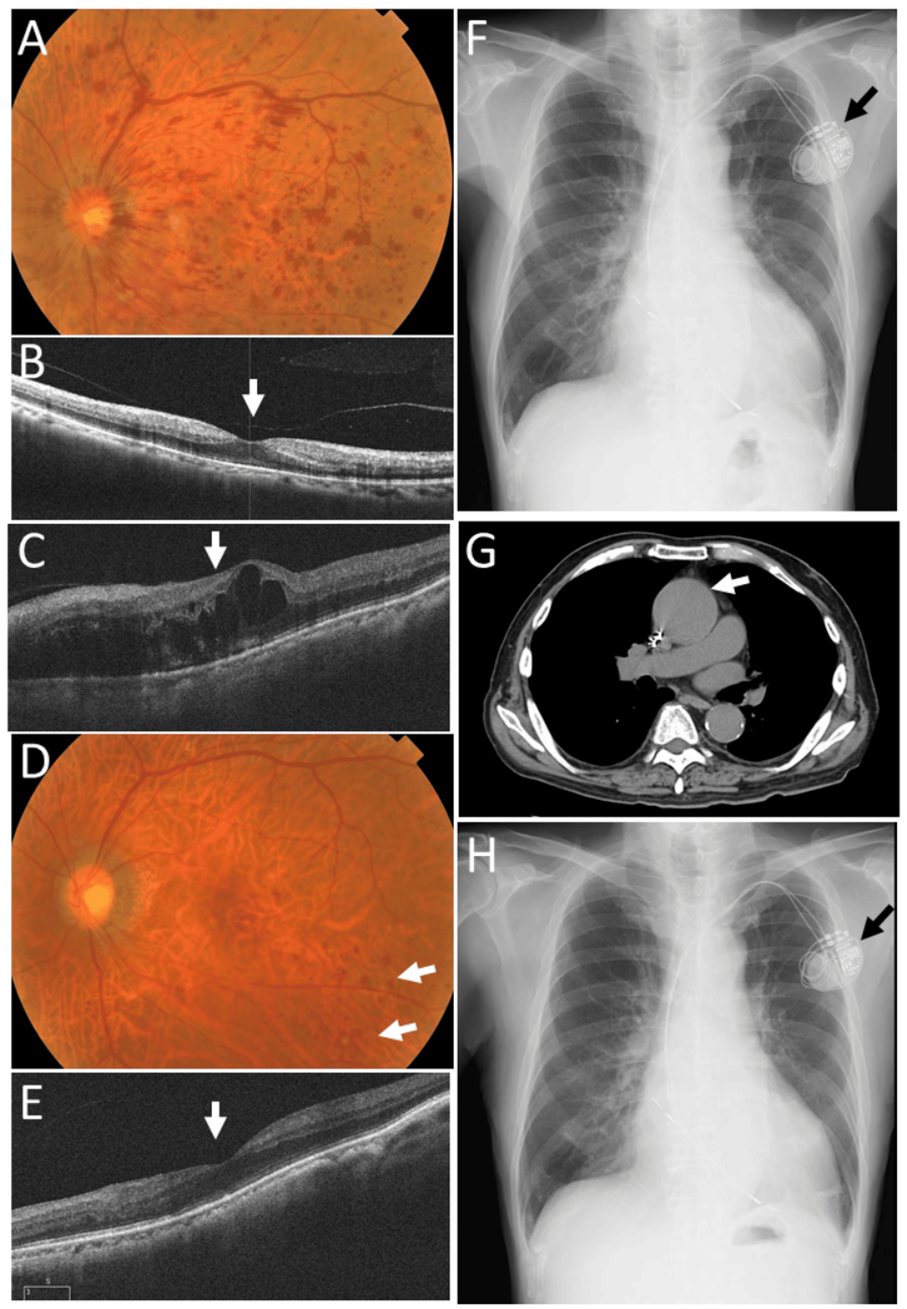
Ophthalmic images, plain chest X-rays, and chest CT at age 72 years Fundus photograph and horizontal OCT image of the left eye at the onset of left CRVO at age 72 years, showing a limited number of flame-shaped and blot retinal hemorrhages with venous dilatation (A) and no macular edema (arrow, B). Horizontal OCT image obtained two months later, immediately after panretinal laser photocoagulation, demonstrating cystoid macular edema (arrow, C). Fundus photograph and horizontal OCT image obtained 10 months later, five months after cardiac surgery, showing a small number of residual blot retinal hemorrhages (arrows, D) and resolution of macular edema (E). Plain chest X-ray films obtained two weeks before the onset of left CRVO (F) and two months later (H) show no change in cardiomegaly, which was greater than that observed one year earlier (Figure 1C, 1D). Chest CT obtained one month later shows no change in the fusiform ascending aortic aneurysm (arrow, G). Black arrows in F and H indicate the heart pacemaker. CRVO, central retinal vein occlusion; OCT, optical coherence tomography

At age 72 years, one year after the initial visit, he developed impending CRVO in the left eye (Figure 3A, 3B). The best-corrected visual acuity was hand motion in the right eye and 0.8 in the left eye. The intraocular pressure was 35 mmHg in the right eye and 7 mmHg in the left eye. After one month, scattered blot retinal hemorrhages and dilated retinal veins in the left eye remained stable. In the following month, he underwent three weekly sessions of panretinal laser photocoagulation in the left eye. Immediately after completion of panretinal photocoagulation, cystoid macular edema developed in the left eye (Figure 3C); however, visual acuity remained 0.8.

Plain chest X-ray films obtained during this period showed no marked change in cardiomegaly (Figure 3F, 3H), which had been enlarged compared with chest X-ray films obtained one year earlier (Figure 1C, 1D). CT demonstrated that the ascending aortic aneurysm remained unchanged, with a maximum diameter of 50 mm (Figure 3G).

During this same period, one month after the onset of CRVO in the left eye, he underwent cardiovascular evaluation to determine surgical eligibility. His blood pressure was 125/72 mmHg. He was 168 cm tall and weighed 56.5 kg. On physical examination, systolic and diastolic murmurs were present, but there was no jugular venous distention or lower extremity edema. Other physical examinations, including neurologic assessment, were unremarkable. Coronary angiography revealed 50% stenosis in segment #3 of the right coronary artery and 50% stenosis in segment #7 of the left anterior descending artery. Left ventriculography demonstrated a preserved ejection fraction of 57% but reduced cardiac output (Table 2) in the presence of mitral valve regurgitation graded as 2-3 (moderate to severe) and aortic valve regurgitation graded as 2-3 (moderate to severe). Swan-Ganz right heart catheterization (Table 2) showed a mildly elevated pulmonary capillary wedge pressure of 16 mmHg (normal range, 6-12 mmHg) and elevated pulmonary artery pressures of 33 mmHg systolic (normal range, 15-30 mmHg), 19 mmHg diastolic (normal range, 8-15 mmHg), and 26 mmHg mean (normal range, 10-20 mmHg).

**Table 2:** Left ventriculography and Swan-Ganz right heart catheterization at age 72 years Normal ranges are provided for reference only and may vary according to age and population [11,12].

Parameter	Normal range	Value	Unit
Pulmonary capillary wedge pressure	6-12	16	mmHg
Pulmonary artery pressure (systolic/diastolic/mean)	15-30/8-15/10-20	33/19/26	mmHg
Right ventricular pressure (systolic/diastolic)	15-25/0-8	29/1	mmHg
Right atrial pressure	2-6	6	mmHg
Cardiac output	4.0-8.0	2.83	L/min
Cardiac index	2.4-4.0	1.8	L/min/m²

**Figure 4:**
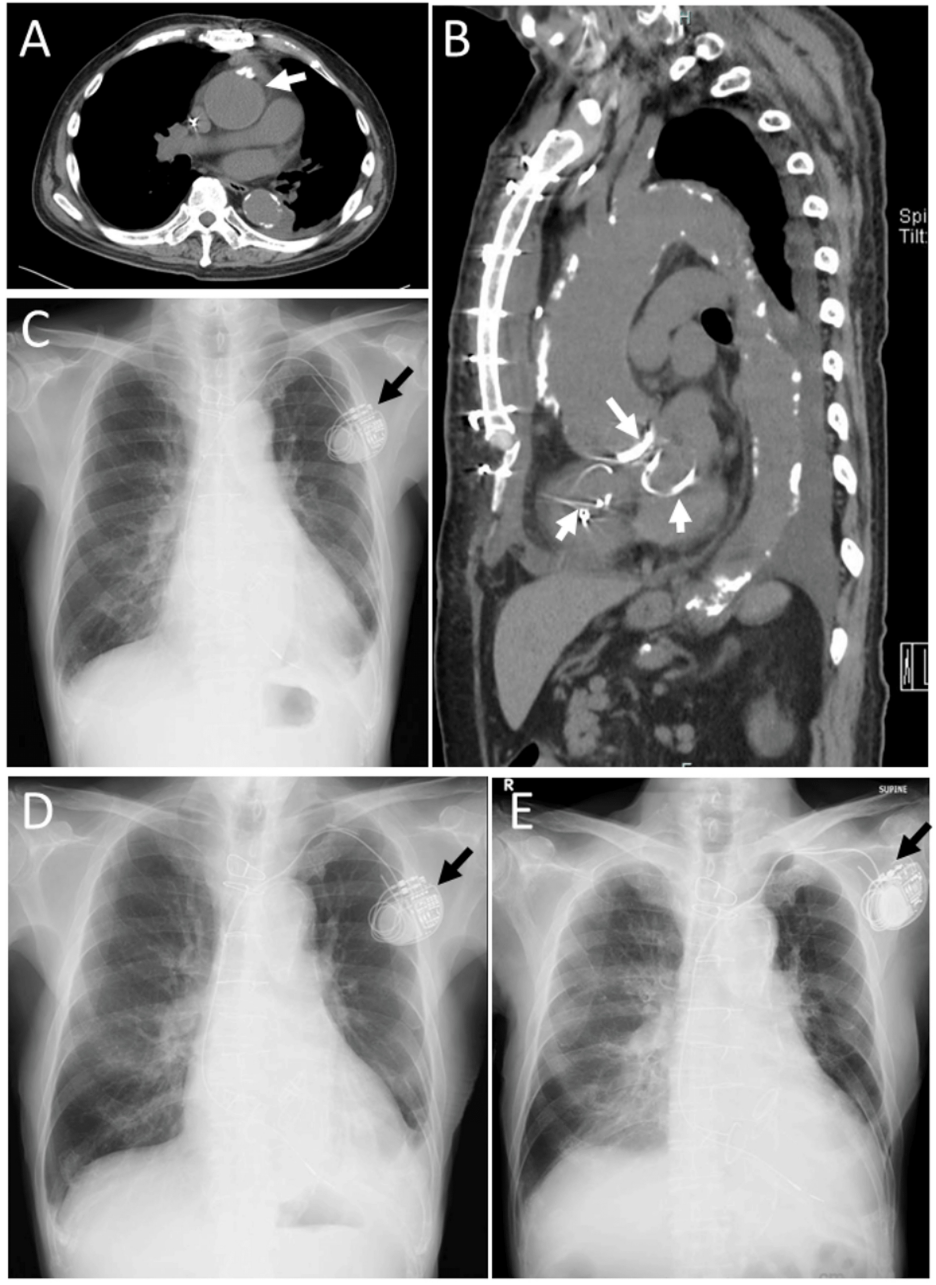
CT scans at age 73 years and plain chest X-rays at ages 73, 80, and 83 years Axial (A) and sagittal (B) CT scans obtained two weeks after cardiac surgery at age 73 years show a fusiform ascending aortic aneurysm with a diameter of 50 mm (arrow, A) and left-sided pleural effusion (A), as well as the aortic valve replacement (arrow, B) and mitral and tricuspid valve annuloplasty (short arrows, B). Plain chest X-ray films obtained one month after surgery (C), at age 80 years (D), and at age 83 years (E), one week before death, demonstrate left-sided pleural effusion in C and D. Marked cardiomegaly with bilateral pulmonary edema in the supine position is noted in E. Black arrows indicate the heart pacemaker.

Five months later, at age 73, he underwent cardiac surgery with cardiopulmonary bypass at a circulation temperature of 32 °C under general anesthesia. The procedures included aortic valve replacement with an SJM bileaflet mechanical aortic valve (23-mm diameter; St. Jude Medical Inc., St. Paul, MN, USA), ascending aortic plication, mitral valve annuloplasty with a MEMO 3D semi-rigid prosthetic ring (32 mm; Corcym Srl, Saluggia, Italy), and tricuspid valve annuloplasty with an MC3 tricuspid annuloplasty ring (28 mm; Edwards Lifesciences, Irvine, CA, USA). Two weeks after surgery, a chest CT showed a fusiform ascending aortic aneurysm with a maximum diameter of 50 mm, and the aortic root diameter had decreased to 42 mm compared with 45 mm before surgery (Figure 4A, 4B). One month after surgery, chest X-ray imaging demonstrated a similar degree of cardiomegaly (Figure 4C). Echocardiography performed four months after surgery showed improved right heart function and stable left heart function, with an ejection fraction of 51% (Table 3). At that time, only a small number of blot retinal hemorrhages without macular edema were observed (Figure 3D, 3E). Visual acuity in the left eye was 0.6.

**Table 3:** Time sequence of echocardiographic indicators and serum BNP levels Normal ranges are provided for reference only and may vary according to age and population [13-16]. BNP, brain natriuretic peptide; CRVO, central retinal vein occlusion; n.d., not determined

Parameter/event	Unit	Normal range	71 years (two months before right-eye CRVO, one month after hemodialysis initiation)	72 years (two months before left-eye CRVO)	73 years (four months after heart surgery)	74 years	76 years	78 years	80 years	81 years	82 years	83 years (three weeks before death)
Left ventricular end-diastolic dimension	mm	35-56	53	57	57	55	57	56	54	53	54	56
Left ventricular end-systolic dimension	mm	20-40	36	39	42	44	43	42	41	38	40	43
Ejection fraction	%	55-70	60	59	51	40	48	49	48	54	50	46
Tricuspid regurgitation pressure gradient	mmHg	Up to 21	28	47	37	24	26	36	32	29	45	52
Inferior vena cava dimension at expiration	mm	9.7-22.6	14	18	15	n.d.	17	n.d.	17	11	17	22
Inferior vena cava dimension at inspiration	mm	4.6-15.4	6	13	8	n.d.	6	n.d.	6	2	4	18
Tricuspid valve regurgitation	-	-	Mild	Moderate	Mild	Mild	Trivial	Mild	Mild	Mild	Moderate	Moderate
Mitral valve regurgitation	-	-	Moderate	Severe	Mild	Mild	Mild	Mild	Moderate	Moderate	Moderate	Moderate
Aortic valve regurgitation	-	-	Moderate	Severe	Mild	Mild	Mild	Mild	Moderate	Moderate	Moderate	Moderate
Pulmonary valve regurgitation	-	-	Trivial	Trivial	Trivial	Mild	Trivial	Trivial	Trivial	Trivial	Trivial	Trivial
BNP	pg/mL	<18.3	709.2	1177	1551.4	n.d.	1114.7	n.d.	n.d.	n.d.	n.d.	735

**Figure 5:**
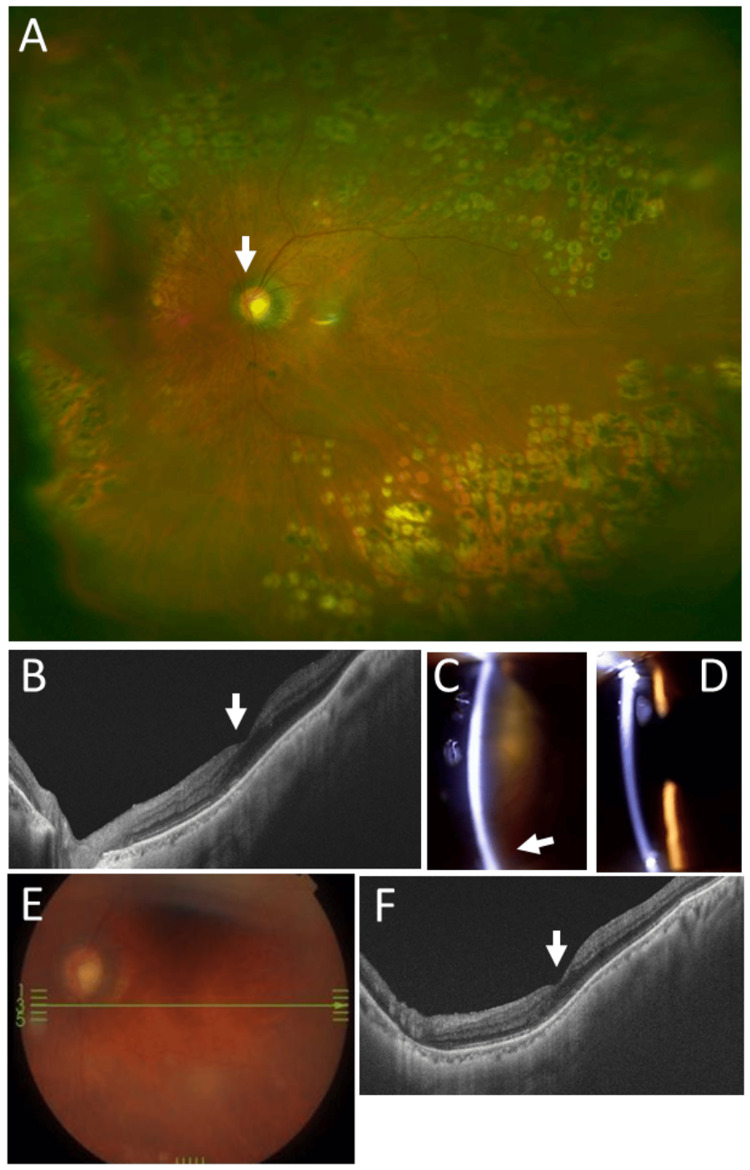
Ophthalmic images at ages 77 and 82 years Wide-field fundus photograph (A) and horizontal section OCT image (B) of the left eye at age 77 years, showing panretinal photocoagulation scars and a normal macula (arrow, B). The arrow in A indicates the optic disc. Slit-lamp photographs of the right eye (C), showing hyphema (arrow) and cataract, and of the left eye (D), showing an intraocular lens. Fundus photograph (E) and horizontal section OCT image (F) of the left eye at age 82 years, one year before death, showing a normal macula (arrow, F). OCT, optical coherence tomography

At age 74 years, he underwent cataract surgery with intraocular lens implantation and achieved a visual acuity of 0.5 in the left eye. At age 77 years, there was no retinal hemorrhage or macular edema in the left eye (Figure 5A, 5B), and visual acuity in the left eye was 0.5. He maintained stable vision in the left eye with well-controlled retinal findings (Figure 5C-5F) and cardiac status (Figure 4D, Table 3) until his final ophthalmologic visit at age 83 years, when the best-corrected visual acuity was no light perception in the right eye and 0.4 in the left eye. Intraocular pressure was 39 mmHg in the right eye and 11 mmHg in the left eye. Mild hyphema was present in the right eye, and no retinal hemorrhage was observed in the left eye, which showed photocoagulation scars. He used topical 0.1% brimonidine twice daily for glaucoma in both eyes. He remained ambulatory and appeared healthy while undergoing hemodialysis three times per week. Two months later, he died of heart failure (Figure 4E).

## Discussion

The present patient was a 71-year-old man who developed CRVO in the right eye and, one year later, CRVO in the left eye. He had undergone heart pacemaker implantation for sick sinus syndrome 10 years earlier and had initiated hemodialysis three months before presentation for chronic renal failure caused by biopsy-unproven hypertensive nephrosclerosis. The right-eye CRVO resulted in neovascular glaucoma and loss of light perception despite intensive treatment with panretinal laser photocoagulation, intravitreal bevacizumab injections, and additional laser therapy. In contrast, the left-eye CRVO remained impending with panretinal laser photocoagulation alone and showed a favorable outcome for 11 years until his death. Retrospectively, he underwent open heart surgery to repair aortic, mitral, and tricuspid valve regurgitation by aortic valve replacement and mitral and tricuspid valve annuloplasty half a year after the onset of left-eye CRVO. Based on the time sequence of events, elevated venous pressure resulting from right heart dysfunction may have been an underlying factor contributing to the poor outcome of the right-eye CRVO, whereas improvement of venous stasis after cardiac surgery may have contributed to the better outcome of the left-eye CRVO.

In general, bilateral CRVO suggests the presence of systemic underlying causes. This patient was a long-term heavy smoker and had hypertension and chronic renal failure. Smoking may exacerbate hypertension and vascular sclerosis and also alter blood coagulation. Chronic renal failure, resulting from persistent hypertension, may further contribute to a hypercoagulable state. Thus, the patient had multiple background risk factors for the development of CRVO [4,5]. He also had a stable fusiform ascending aortic aneurysm and sick sinus syndrome with pacemaker implantation, as well as aortic, mitral, and tricuspid valve regurgitation. At the initiation of hemodialysis, his cardiac condition was evaluated by echocardiography two months before the onset of right-eye CRVO. Over the course of the sequential development of right- and left-eye CRVO with a one-year interval, echocardiography demonstrated deterioration of right heart function, mainly due to tricuspid valve regurgitation (Table 3). One month after the onset of left-eye CRVO, an overall increase in right-sided cardiac pressures and reduced left ventricular cardiac output led to the recommendation for surgical intervention [11,12]. Follow-up echocardiographic examinations showed improved right heart parameters in the years after successful surgery for aortic, mitral, and tricuspid valve regurgitation [13-16] (Table 3).

Considering these systemic conditions, the poor outcome of the right-eye CRVO occurred during a period of worsening right heart function, whereas the better outcome of the left-eye CRVO coincided with improved right heart function. Notably, the patient had “moderate” tricuspid valve regurgitation at the time of right-eye CRVO, whereas he consistently showed “mild” tricuspid valve regurgitation after cardiac surgery until the age of 81 years (Table 3). In a similar context, bilateral CRVO has been reported in a patient with Eisenmenger syndrome, which is characterized by elevated right heart pressures secondary to congenital heart defects such as atrial or ventricular septal defects [17]. Additionally, increased episcleral venous pressure with advanced glaucoma has been described in association with Eisenmenger syndrome [18]. Taken together, these observations suggest that elevated central venous pressure may represent a background condition for the development of CRVO, as illustrated in the present case.

The central retinal vein drains into the ophthalmic vein and subsequently into the cavernous sinus. In this setting, cavernous sinus thrombosis [19,20] and carotid-cavernous fistula [21] have been reported as causes of CRVO. The present patient did not show pulsatile exophthalmos, a clinical sign suggestive of carotid-cavernous fistula, and had no abnormal findings on head CT or MRI. The right eye exhibited more severe CRVO than the left eye. From another perspective, the right-sided venous system is anatomically closer to the superior vena cava and may therefore be more susceptible to increased right heart pressures. A major limitation of attributing the findings solely to right heart dysfunction is that the patient was receiving hemodialysis three times weekly, which induces marked fluctuations in body fluid volume. Although echocardiography (Table 3) was performed on non-dialysis days, measurements were inevitably influenced by volume status. Moreover, it is noteworthy that the patient did not demonstrate jugular venous distention during the clinical course, which may also be attributable to fluid volume changes associated with hemodialysis.

At the time of cardiac surgery, when the patient was 73 years old, he was physically stable on hemodialysis (Table 1). Based on recommendations from both a nephrologist and a cardiologist, he underwent open heart surgery to halt progressive deterioration of both right and left heart function by repairing aortic, mitral, and tricuspid valve regurgitation (Table 2, Table 3). In the absence of other risk factors, such as periodontitis, for valvular disease [22], the combined valvular dysfunctions were attributed to cardiac enlargement associated with the ascending aortic aneurysm, which itself was likely a sequela of long-standing hypertension. Chronic renal failure in this patient was also likely caused by hypertension. Under these circumstances, simultaneous repair of the aortic, mitral, and tricuspid valves was planned by the cardiovascular surgeon [23]. For 10 years after successful cardiac surgery (Table 3), the patient remained clinically stable on hemodialysis three times weekly until his death. He also maintained ambulatory vision in the left eye, which had a favorable outcome after CRVO, likely owing to improvement of venous stasis following cardiac surgery. It should again be noted, as a limitation of this interpretation, that hemodialysis inherently induces changes in body fluid status and intraocular pressure. Nevertheless, the clinical course of this patient suggests that venous stasis, in addition to coagulopathy, may be an important background factor in the development of bilateral CRVO [24].

## Conclusions

This report describes an elderly patient who developed bilateral CRVO sequentially, first in the right eye and then in the left eye, with a one-year interval. The right-eye CRVO was uncontrollable despite intensive panretinal laser photocoagulation combined with intravitreal bevacizumab, resulting in no light perception. In contrast, the left-eye CRVO, which occurred one year later, remained impending and had a favorable outcome with panretinal laser photocoagulation after the patient underwent open heart surgery to repair tricuspid, mitral, and aortic valve regurgitation. Elevated venous pressure due to right heart dysfunction may have contributed to the poor outcome in the right eye, whereas surgical correction of venous pressure likely facilitated the favorable outcome in the left eye. This case highlights that venous stasis, in addition to coagulopathy, may serve as an important underlying factor in the development of bilateral CRVO.
